# Developing novel Lin28 inhibitors by computer aided drug design

**DOI:** 10.1038/s41420-024-02281-z

**Published:** 2025-01-12

**Authors:** Victor M. Matias-Barrios, Mariia Radaeva, Graciella Rosellinny, Qiongqiong Jia, Ning Xie, Monica Villanueva, Hanadi Ibrahim, Jason Smith, Martin Gleave, Nada Lallous, Suzana K. Straus, Artem Cherkasov, Xuesen Dong

**Affiliations:** 1 The Vancouver Prostate Centre, Department of Urologic Sciences, University of British Columbia, 2660 Oak Street, Vancouver, BC V6H 3Z6 Canada; 2 Department of Chemistry, University of British Columbia, 2036 Main Mall, Vancouver, BC V6T 1Z1 Canada

**Keywords:** Drug discovery and development, Virtual screening

## Abstract

Lin28 is a key regulator of cancer stem cell gene network that promotes therapy-resistant tumor progression in various tumors. However, no Lin28 inhibitor has been approved to treat cancer patients, urging exploration of novel compounds as candidates to be tested for clinical trials. In this contribution, we applied computer-aided drug design (CADD) in combination with quantitative biochemical and biological assays. These efforts led to the discovery of Ln268 as a drug candidate that can block Lin28 from binding to its RNA substrates and inhibit Lin28 activities. Ln268 suppressed Lin28-mediated cancer cell proliferation and spheroid growth. Results from nuclear magnetic resonance spectroscopy confirmed that Ln268 perturbs the conformation of the zinc knuckle domain of Lin28, validating the rational drug design by CADD. The inhibitory effects of Ln268 are dependent on Lin28 protein expression in cancer cells, highlighting limited off-target effects of Ln268. Moreover, Ln268 synergizes with several chemotherapy drugs to suppress tumor cell growth. In summary, Ln268 is a promising candidate for further development to target Lin28 as a cancer therapy.

## Introduction

The RNA-binding protein Lin28, along with three other transcription factors (Sox2, Oct4, and Nanog), comprise the four core stemness gene network [1]. When combined, these genes have the ability to transform terminally differentiated fibroblasts into inducible pluripotent stem cells [1]. The requirement of all four genes to induce pluripotency suggests that their signaling pathways are interconnected, and disruption of one gene can interfere with the others in the stemness gene network. Indeed, Lin28 is known to regulate the expression of Sox2, Oct4, and Nanog in various cancer cells [2–4]. Consistently, we reported that Lin28 regulates Sox2 expression through HMGA2 in therapy-resistant neuroendocrine prostate cancer cells. Depletion of Lin28 by either siRNA or CRISPR blocks the cancer stem cell (CSC) gene signature, cancer cell growth, xenograft formation, and progression [4]. While Sox2, Oct4, and Nanog are transcription factors that are notoriously difficult to block with small molecule inhibitors, focusing on inhibiting Lin28 to prevent its binding to RNA substrates emerges as an attractive strategy for cancer therapy [5, 6].

Human cells have two Lin28 genes (Lin28a and Lin28b) that are highly homologous but are not typically co-expressed in the same cell lineage [7]. Both genes are commonly not expressed in fully differentiated benign cells in adults but exhibit low levels of expression in stem cells from the placenta and testis. However, Lin28a or Lin28b is frequently overexpressed in primary tumors and, more significantly, in therapy-resistant tumors [3]. Their enhanced expression is associated with high tumor metastasis rate and poor patient prognosis [6]. While the microRNA let-7 is a well-established tumor suppressor, the oncogenic role of Lin28 is best characterized as a let-7 inhibitor; Lin28 binds to let-7 microRNA precursors and further recruits the terminal uridylyl-transferases (TUTs) to initiate polyuridylation modification of pre-let-7 and subsequently pre-let-7 degradation [8]. Lin28 has a cold shock domain (CSD) and a zinc knuckle domain (ZKD) that bind pre-let-7 at the GNGAY and GGAG RNA motifs, respectively [9, 10]. Although the primary role of Lin28 is known to regulate let-7 synthesis, recent studies have revealed several additional functions: (1) Lin28’s RNA binding ability also influences mRNA translation; it can bind mRNAs such as Oct4 and further recruit RNA Helicase A to enhance Oct4 translation [11–14]. (2) Lin28 can be localized in P bodies, where it plays a role in RNA degradation [15]. (3) Under stress conditions, Lin28 clusters with G3BP1 and YB-1 in stress granules (SGs) to protect mRNAs from being translated [16], and (4) Lin28 was reported to regulate M6A methylation of mRNAs to impact gene expression [17]. These findings indicate that pharmacologically interrupting Lin28-RNA interactions would be detrimental to Lin28 functions.

Despite widespread consensus regarding the potential benefits of Lin28 inhibitors for cancer patients, efforts to develop small molecule inhibitors targeting Lin28 have not resulted in any inhibitors advancing to clinical applications [6]. The majority of studies have employed a high-throughput screening strategy utilizing fluorescence resonance energy transfer (FRET) or fluorescent polarization (FP) assays [6]. While several compounds have been identified, they exhibit low potency and, more importantly, often lack a clearly defined mode of action (MOA). For example, C1632 demonstrates inhibitory effects on cancer cells only at concentrations exceeding 100 μM [18]. Furthermore, there is insufficient information available regarding the structure-activity relationship (SAR) between C1632 and the Lin28 protein, hindering further optimization of this compound.

In this study, we employed computer-aided drug design (CADD) to conduct rational drug design by leveraging the existing crystal structure of the Lin28:pre-let-7 complex [19]. Several candidate compounds were designed from existing scaffolds to target a previously validated drug docking pocket at the interface between Lin28 ZKD and pre-let-7 miRNA. We found that the compound Ln268 is a potent Lin28 inhibitor, obstructing Lin28 RNA binding capability. Ln268 exhibited robust inhibition of cancer stem cell (CSC) phenotypes and tumor spheroid growth. Furthermore, it demonstrated synergistic effects to suppress tumor cell growth when co-administered with chemotherapy drugs. These results underscore the capability of CADD in developing novel Lin28 inhibitors as potential anticancer agents.

## Results

### Design novel Lin28 inhibitors by CADD

Our rational drug design aims to block the ZKD from binding to the GGAG RNA motif of let-7, as it is the ZKD-GGAG interaction that initiates the recruitment of TUTs to degrade let-7 precursors [20]. All let-7 members suppressed by Lin28 contain the GGAG motif, and substitutions of G1 and G2 with A (GGAG to AGAG or GAAG) abolish Lin28’s capacity to degrade let-7. The ZKD also hosts a highly druggable site that engages G1 and A3 of the GGAG motif [20]. Mutations at Y140 and H148 within this pocket block Lin28-mediated let-7 degradation [21]. Using this docking site, we found several compounds that can block Lin28 activities [22], providing proof-of-concept to further develop small molecule inhibitors to interrupt ZKD-GAGG interactions.

**Fig. 1:**
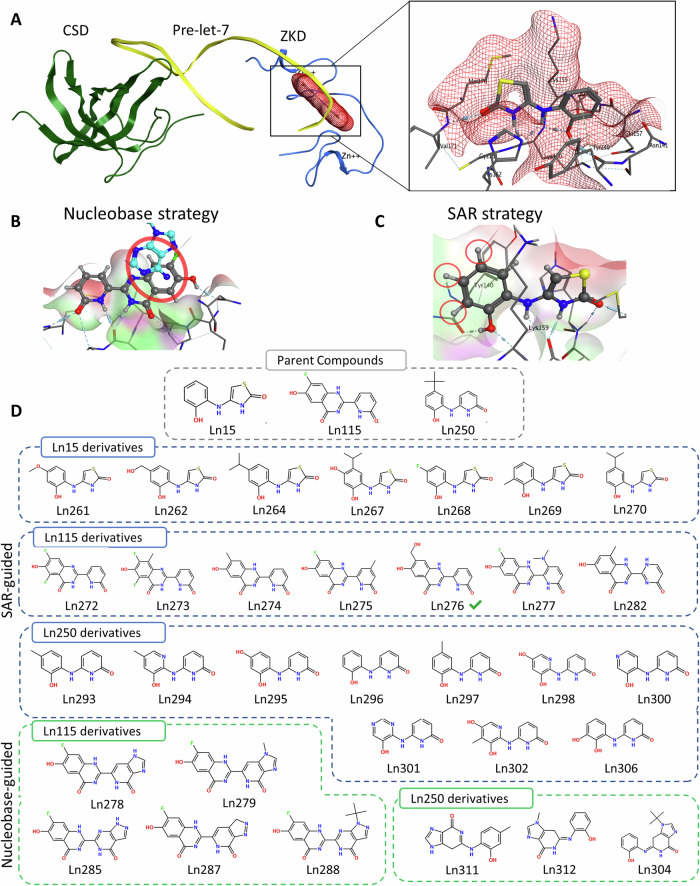
Lin28 Inhibitor derivatives design strategies. **A** Spatial relationship of Lin28 ZKD, pre-let-7 RNA and docking pocket. **B** Structure-activity relationship guided design. Compounds Ln15 and Ln7 were previously modified, and the derivatives were tested. Active derivatives Ln245 and Ln250 indicate that hydrophobic moieties might be beneficial for pre-let-7 binding inhibition. **C** Nucleobase-inspired design. Compound Ln115 overlaid with the guanine and adenosine residues of pre-let-7 GGAG motif. The modification to hydroxupurine is highlighted in red. **D** Compounds developed by SAR and nucleobase-based designs.

We applied both nucleobase-inspired design and SAR guided design approaches to develop new Lin28 inhibitors. In the nucleobase-inspired design, we modified previously reported compounds (Ln7, Ln15, and Ln115) to resemble the nucleoside or nucleobase of the GGAG motif natively bound to Lin28. This strategy was successful in the past to design nucleoside or nucleobase analogs as antiviral and anticancer drugs [23, 24]. We overlaid our lead compounds with G1 and A3 of the GGAG motif using the Lin28-let-7 crystal structure (PDB: 5UDZ [19]) (Fig. 1A, B). Then, we added structural modifications to the lead compounds to achieve higher similarity between the selected compounds and the nucleosides. For example, the hydroxypyridine ring of Ln115 was modified into various analogs of hydroxyopurines to resemble the G1. Similarly, compound Ln250 (an analog of Ln7) was modified by adding an imidazole ring to resemble the purine base of the G1. We also added a sugar moiety to the modified Ln250 structure to replicate the G1-Lin28 interaction. In the SAR guided design, we used data collected from FP assays testing Ln7, Ln15, Ln115, and their analogs to determine several structure-activity relationship patterns (Fig. 1C). For instance, Ln245, a close analog of Ln15 with methyl and isopropyl groups added to the phenol ring, showed relatively higher potency than Ln15. Ln250, with a tetrabutyl attached to the phenol ring, also outperformed Ln15. These results suggested that large hydrophobic moieties placed on the phenol ring could enhance the activity of newly designed compounds. We designed several analogs of Ln7, Ln15, and Ln250 by adding hydrophobic methyl, ethyl, isopropyl, and tetrabutyl groups. In addition, we designed several chimera compounds by combining features of Ln15 and Ln250. For instance, we replaced the thiazole ring of compound Ln15 with a bulkier thiazine ring that closely resembles the binding mode of Ln250.

All selected compounds underwent evaluation with molecular docking software and multiple filtration algorithms to ensure that the candidate compounds surpassed their parental compounds in docking performance. To evaluate the efficacy of the scoring metrics, we correlated the obtained scores with the experimental activity of previously identified compounds [22]. The two key metrics that most accurately distinguished active from inactive compounds were Molecular Mechanics Generalized Born Surface Area (MM-GBSA) ligand efficiency (R = −0.44) and Glide docking ligand efficiency (R = −0.53). The MM-GBSA method is used to estimate binding free energies of ligands and is generally considered a more accurate method than docking [25]. All newly designed analogs were assessed with these scoring methods, and only compounds that exhibited better scores than their parent compounds were selected for further testing. In addition, all compounds were docked using Glide [9], ICM [26], and FRED [27] docking software to ensure reliable predicted binding poses (i.e., to check for consensus poses among docking software). Finally, the compounds underwent screening with an absorption, distribution, metabolism, excretion, and toxicity (ADMET) predictor platform to ensure metabolic safety. All selected compounds exhibited an acceptable ADMET profile, and the docking poses did not significantly differ between the docking programs (i.e., RMSD < 3 Å). To this end, 32 compounds were certified and custom synthesized by Life Chemicals Ltd (Fig. 1D). Upon receiving these compounds, LC/MS/MS was used to confirm the purity and the molecular masses.

### Biochemical validation of the designed compounds

**Fig. 2:**
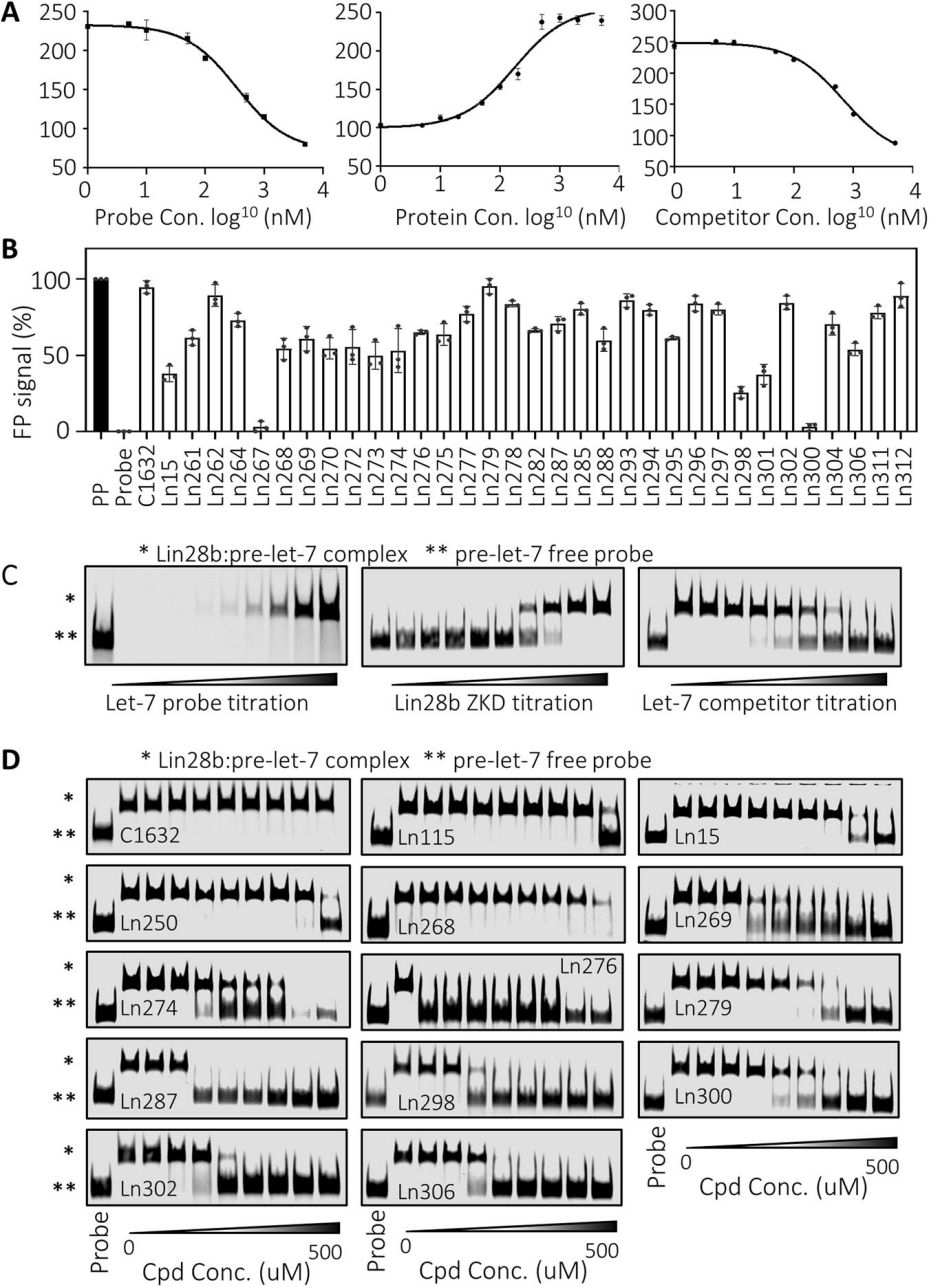
Validate CADD predicted compounds by biochemistry assays. **A** Establish optimal FP assays by titrating the Lin28b ZKD protein (left), fluorescent labeled pre-let-7 RNA probe (middle), and non-fluorescent labeled pre-let-7 RNA probe. **B** FP assays screened all 32 compounds designed by CADD. **C** Establish optimized conditions for EMSA assays using Lin28b ZKD and fluorescent labeled pre-let-7 RNA probe. **D** increasing doses of 13 Lin28b inhibitors identified by FP assays were used in the optimized EMSA assays. Three independent biological replicates were performed for all the assays. All results are presented as the mean ± SD. Only one representative EMSA image was shown for each compound.

We conducted two biochemical assays, FP and EMSA, to validate the predicted compounds. To optimize the FP assays, we performed a series of titrations using: (1) FAM-labeled pre-let-7d RNA probe, (2) Lin28b ZKD protein, and (3) non-labeled pre-let-7 probe as a competitor (Fig. 2A). These assays yielded the optimal concentrations required for the subsequent FP validation assays, namely 10 nM for the pre-let-7d probe and 1 μM for Lin28b ZKD protein. The FP signal from the free probe was set as 0%, while the signal from the probe-ZKD complex in the absence of compounds was set as 100%. C1632 was used as a control Lin28 inhibitor (Fig. 2B). Out of the 32 compounds tested, 13 showed at least 40% inhibition of the Lin28b ZKD-let-7 interaction. Furthermore, all 13 compounds exhibited dose-dependent inhibition of Lin28b ZKD:pre-let-7 interactions (Figure S1). We observed that Ln267 and Ln302 had IC_50_s of 4.67 μM and 3.63 μM, respectively, which were lower than the parent compounds Ln15 and Ln115 with respective IC_50_s of 9 and 21 μM (Table S1). Since the docking pocket is almost identical between Lin28a and Lin28b homologs, we repeated the FP assays using Lin28a ZKD (Figure S2). We found that at least 8 compounds (Ln267, Ln272, Ln279, Ln287, Ln298, Ln300, Ln302, and Ln306) were more potent than Ln15 and Ln115 in blocking the Lin28a ZKD:pre-let-7 interaction. These results indicate that many of these newly designed compounds have improved potency in disrupting pre-let-7 interactions with ZKD from both Lin28a and Lin28b.

We next validated the newly developed compounds using EMSA, a non-florescent-based assay to detect Lin28b ZKD:pre-let-7 complex formation. Similar to the FP assays, we first optimized the experimental conditions by titrating: (1) IRDye800-labeled pre-let-7d RNA probe, (2) Lin28b ZKD protein, and (3) non-labeled pre-let-7 probe as a competitor (Fig. 2C). For the validation experiments, 1 μM Lin28 ZKD and 10 nM of IRDye800-labeled pre-let-7d probe were mixed with each candidate compound. Compounds C1632 and Ln15 were used as control Lin28 inhibitors, respectively. While the 13 compounds validated by FP assays showed inhibition of the ZKD:pre-let-7 complex, EMSA confirmed that 10 compounds (Ln268, Ln269, Ln274, Ln276, Ln279, Ln287, Ln298, Ln300, Ln302, and Ln306) were capable of inhibiting Lin28:pre-let-7d complex formation in a dose-dependent manner (Fig. 2D). The discrepancy between FP and EMSA results may be attributed to the different sensitivities of each assay [28] or the compounds had profound impacts to fluorescence signals that are technically difficult to demonstrate. Regardless, we use these two biochemical assays to shortlist the most promising compounds for further characterization. It is worth noting that we did not observe any obvious inhibition of C1632 on either the Lin28a or Lin28b protein using the same EMSA conditions as those used for testing our candidate compounds. This indicates that our Lin28 inhibitors have different MOAs from C1632. While our inhibitors directly target the Lin28 ZKD, it is unclear whether C1632 may bind to domains other than the ZKD or allosterically disrupt Lin28’s capacity to bind its RNA substrates. Together, these results showed that the thirteen candidate compounds were validated by either FP or EMSA to be Lin28 inhibitors.

### Biological validation of the designed compounds

**Fig. 3:**
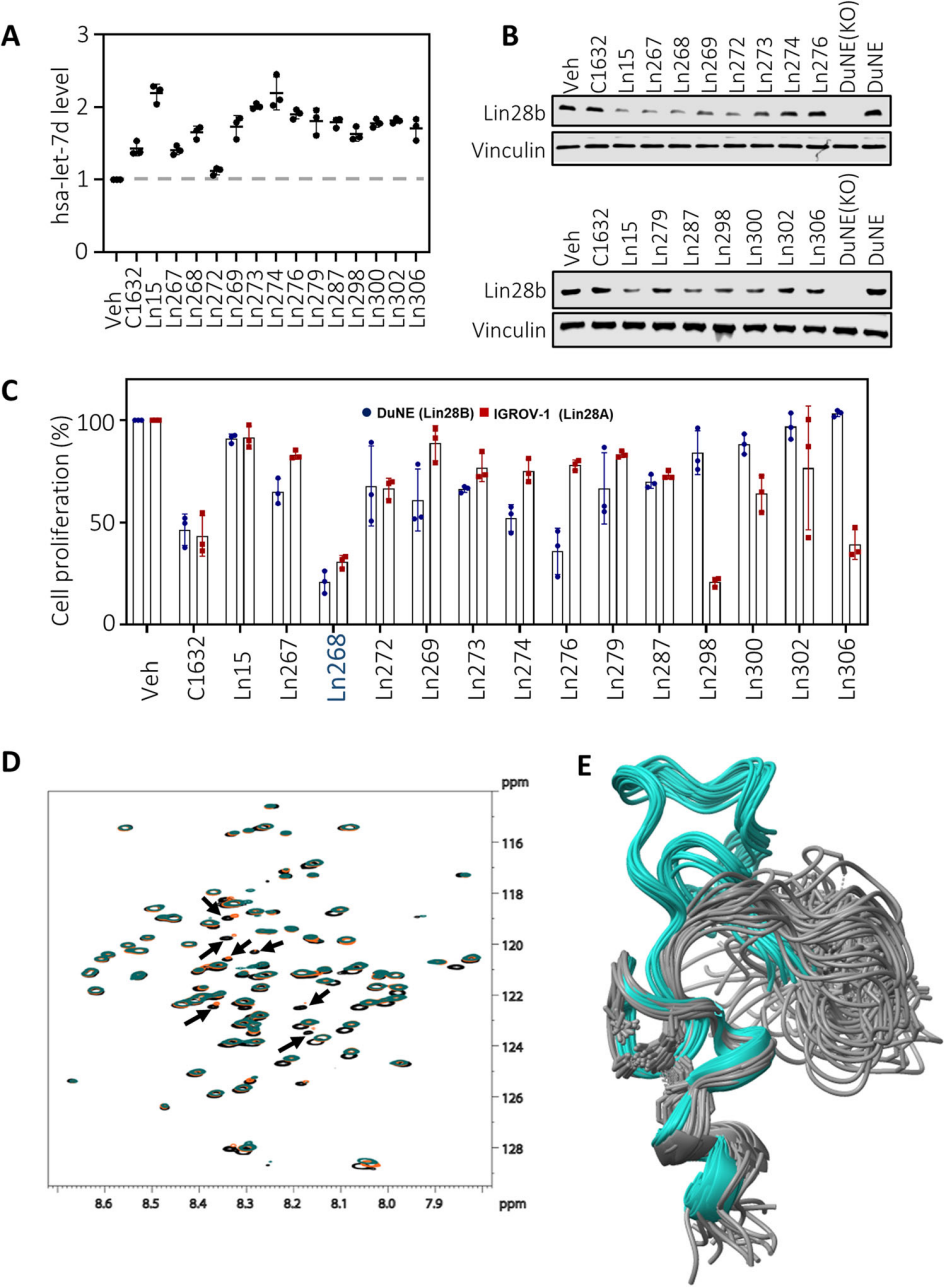
Validate CADD predicted compounds by cell-based assays. **A**, **B** DuNE cells were treated with Lin28b inhibitors for 72 h. Let-7d RNA levels were measured by real-time qPCR (**A**) and Lin28b protein levels were measured by immunoblotting (**B**). **C** Lin28b-positive DUNE cells and Lin28a-positive IGROV-1 cells were treated with Lin28 inhibitors. Cell proliferation rates were measured by Incucyte. **D** Overlay of 1H-15N HSQC spectra for Lin28b-ZKD (black), Lin28b-ZKD + Ln15 (orange), and Lin28b-ZKD + Ln268 (blue). Ligands were added in a 20:1 molar ratio. Approximately 20 residues are seen to shift (from black to orange/blue), with ca. 5 resonances disappearing for Lin28b-ZKD in the presence of Ln268. **E** Overlay of the 20 NMR models for 2CQF (gray) and 2LI8 (blue) showing the rearrangement of residues 137–160 in going from Lin28b-ZKD alone to the conformation with RNA present. The conformation of Lin28b-ZKD in the presence of the ligands described herein might resemble that of 2LI8, but with a larger conformational spread. All results are presented as the mean ± SD.

We further tested whether the 13 candidate compounds could penetrate the cell membrane, endure cell-mediated metabolism and excretion, and inhibit Lin28 activities inside cancer cells. Lin28b-positive DuNE cells [29] were used in this experiment. Since let-7 miRNA is expressed at low levels in DuNE cells due to high Lin28b expression, all compounds blocked Lin28b mediated suppression of let-7 levels by 11 to 210% (Fig. 3A). With the exception of Ln267 and Ln272, all compounds showed stronger capacity to increase let-7 expression than C1632. Since let-7 and Lin28b mutually regulate each other’s expression, increased let-7 expression by Lin28 inhibitors would suppress Lin28b protein levels. We found that 8 (Ln267, Ln268, Ln269, Ln272, Ln273, Ln287, Ln298, and Ln300) out of the 13 compounds at a concentration of 20uM suppressed Lin28b protein expression (Fig. 3B). Although multiple compounds can block the molecular functions of Lin28b, Ln268 was the only compound that strongly inhibited cell proliferation in both Lin28b-positive DuNE cells and Lin28a-positive IGORV-1 cells (Fig. 3C). Furthermore, these suppressive effects were more potent than the parent compound Ln15. As a consequence of these results, Ln268 was identified as the lead Lin28 inhibitor for further characterization.

### Comparison of the effect of Ln15 and Ln268 on the conformation of Lin28 ZKD

Given that Ln268 is derived from the parent compound Ln15, we wanted to assess whether the improved properties of Ln268 were linked to a potential difference in its interaction with Lin28 ZKD. To this end, we collected ^1^H-^15^N HSQC spectra of Lin28b-ZKD, Lin28b-ZKD + Ln15, and Lin28b-ZKD + Ln268. RNA binding proteins (RBPs) commonly have binding pockets that are intrinsically disordered [30], as evidenced by the narrow chemical shift dispersion observed in the HQSC of Lin28b-ZKD alone (Fig. 3D). Addition of either Ln15 or Ln268 led to changes in the chemical shifts of ca. 20 residues, consistent with the change in conformation of the Lin28b-ZKD segment which encompasses residues 137–160 (three of which are prolines) upon RNA binding (Fig. 3E). Although many of the peak shifts are the same for the Ln15 and Ln268 containing samples, there are around 7 resonances where the peaks disappear in the case of Ln268. This suggests that the conformation of Lin28b-ZKD is slightly different in the presence of Ln15 versus Ln268.

### Biological characterization of Ln268 as a Lin28 inhibitor

**Fig. 4:**
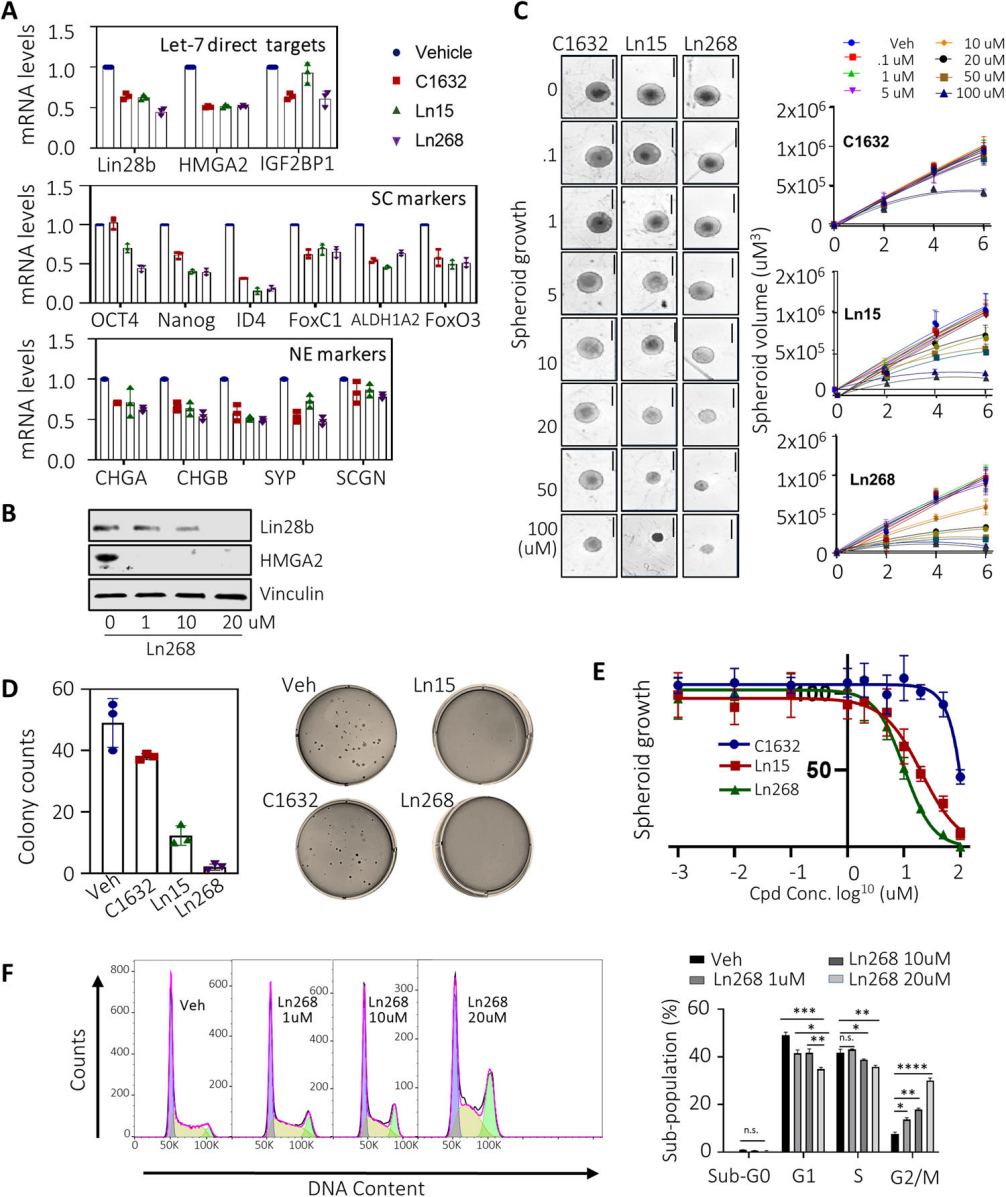
Ln268 strongly blocks cancer cell stemness of t-NEPC cells. **A**, **B** DuNE cells were treated with C1632, Ln15, and Ln268 for 72 h. Selected Let-7 targeted genes, stem cell biomarkers and NE biomarkers were measured by real-time qPCR (**A**). Lin28b and its downstream HMGA2 protein levels were measured by immunoblotting (**B**). **C** DuNE spheroids were treated with 0–100 μM of C1632, Ln15, and Ln268 for 0–6 days. Spheroid sizes were measured and plotted. **D** DuNE cells were used to perform colony formation assays in the presence of C1632, Ln15 and Ln268 for 14 days. The number of colonies formed in each treatment counted and plotted. **E** DuNE cells under 3D culture conditions were treated with C1632, Ln15, and Ln268 at the indicated concentrations. Cell growth rates were measured by Incucyte. **F** DuNE cells were treated with 0–20 μM Ln268 for 144 h. FACS measured cell populations at each stage of cell cycling. Three independent biological replicates were performed for all the assays. All results are presented as the mean ± SD.

We previously demonstrated depletion of Lin28b in DuNE cells using either RNA silencing or CRISPR suppressed let-7 target genes, as well as several CSC and Neuroendocrine (NE) biomarkers [4]. When DuNE cells were treated with Ln268, C1632, or Ln15, we found that Ln268 potently inhibited Lin28b at both mRNA and protein levels (Figs. 4A, B, S6) as well as a panel of genes that are direct let-7 targets, CSC, and NE biomarkers, including HMGA2, IGF2BP1, Oct4, Nanog, ID4, FoxC1, ALDH1A2, FoxO3, CHGA, CHGB, SYP, and SCNG. In the cases of Oct4, Nanog and ID4, Ln268 showed stronger inhibitory effects than C1632. These results indicated that Ln268 inhibits Lin28b activities in DuNE cells, leading to the suppression of CSC and NE phenotypes.

CSCs have characteristic tumorigenic, self-renewal, and differentiation properties [31]. The self-renewal ability is hijacked by CSCs that makes them both tumorigenic and capable of sustaining long-term tumor growth, which can be evaluated by 3D spheroid and colony formation assays. We established a DuNE spheroid model in which DuNE cells formed 3D spheroids in 96-well plates (Figure S3). By integrating this 3D culture with the Incucyte live-cell imaging system, we quantified the sizes of the spheroid growth over 0–6 days to generate a spheroid growth curve. Using this model system, we treated the DuNE spheroids with increasing doses (0–100 μM) of C1632, Ln15, and Ln268 for 0–6 days. We found that to achieve 50% spheroid growth inhibition, it required 100 μM of C1632, 50 μM of Ln15, but only 10 μM of Ln268 (Fig. 4C). These results indicate that Ln268 has a potency approximately 10-fold stronger than C1632 and a ~2-fold improvement over its parent compound Ln15. Ln268 exhibited the strongest potency among all 13 candidate compounds that tested positive in biochemical assays (Figure S4). In addition, classic colony formation assays were used to measure the stemness in the DuNE model. Colony formation counts, which were ~50 under vehicle treatment after 14 days, decreased by 22% in C1632-treated plates, and by 75% and 96% in the Ln15 and Ln268 treated plates, respectively (Fig. 4D). These results demonstrate that Ln268 strongly inhibits the CSC phenotype of cancer cells.

To further compare the potency of Ln268 with C1632 and Ln15 in suppressing DuNE cell growth, we also measured cell growth rates under 2D conditions (Fig. 4E). Consistent with our previous findings, C1632 had no inhibitory effects at concentrations below 100 μM, while both Ln15 and Ln268 showed dose-dependent inhibition of cell growth starting at 5 μM (Fig. 4F). Ln268 exhibited stronger suppressive effects than Ln15. In addition, we used FACS assays to confirm that Ln268-mediated cell growth inhibition was due to its effects on blocking cancer cell cycling from the G1 phase towards the S and M phases (Fig. 4F). After 144 h treatment, cells were arrested in G2/M in a dose-dependent manner. In summary, we confirmed that Ln268 is a potent Lin28 inhibitor that blocks the CSC phenotype of cancer cells, suppresses formation of 3D colonies, and inhibits cancer cell proliferation.

### Ln268 has limited off-target effects

**Fig. 5:**
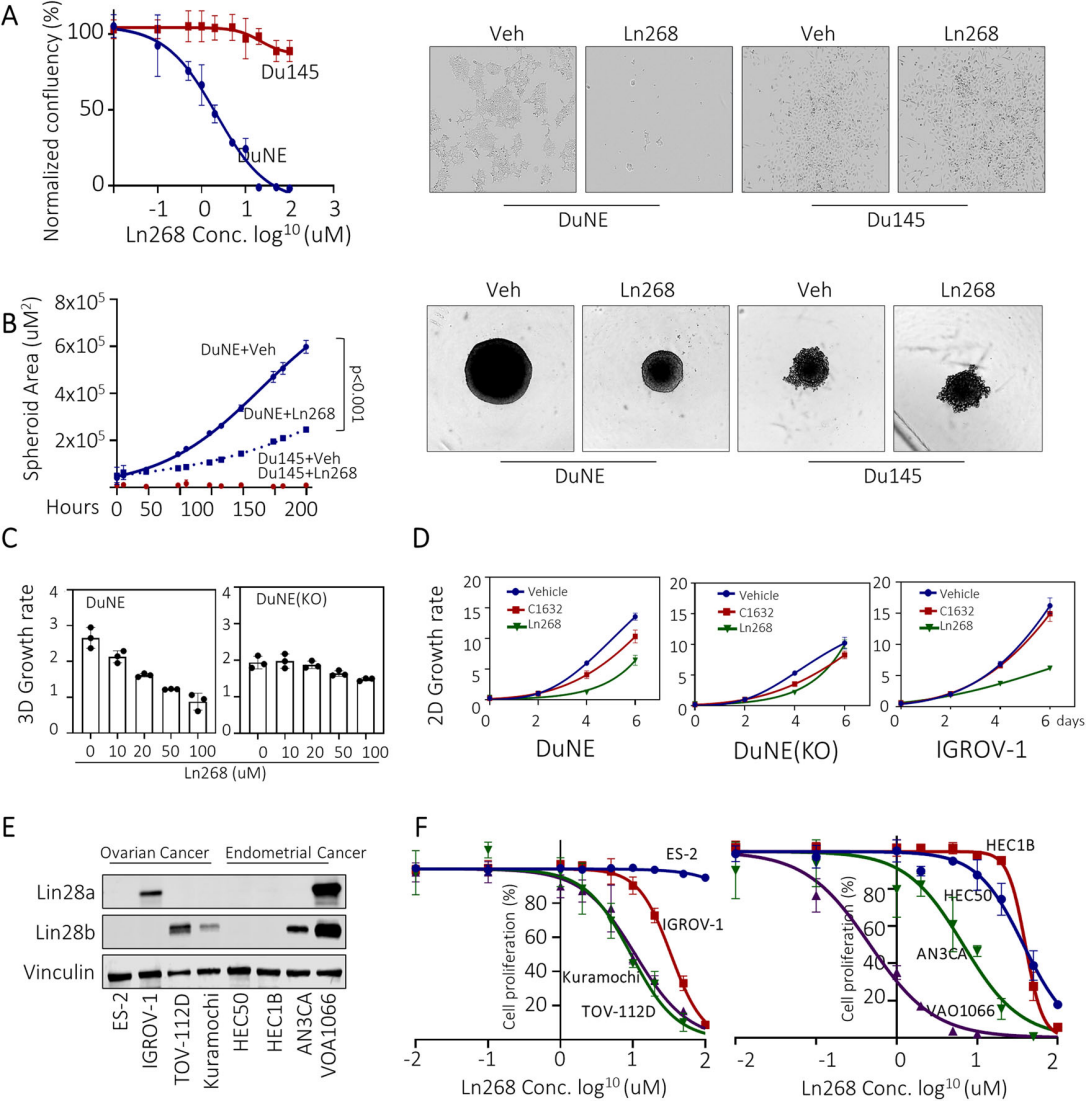
Limited off-target effect of Ln268. **A** Lin28 positive DuNE cells and Lin28 negative DU145 cells were treated with increasing doses of Ln268. Cell proliferation rates were measured by Incucyte. Cell morphology was also captured under control or Ln268 treatment. **B** Lin28 positive DuNE cell spheroids and Lin28 negative Du145 cell spheroids were treated with Ln268 for 0–192 h. Spheroid sized were measured and plotted. **C** Lin28b-positive DuNE cells, DuNE cell with Lin28b knockout were treated with increasing doses of Ln268 for 72 h. Cell proliferation rates were measured and plotted. **D** Lin28b-positive DuNE cells, DuNE cell with Lin28b knockout and Lin28a-positive IGOV-1 cells were treated with control C1632 and Ln268 for 0–6 days. Cell proliferation rates were measured by Incucyte and plotted. **E** Immunoblotting assays measured Lin28b and Lin28a protein expression in several ovarian and endometrial cancer lines. **F** Ovarian and endometrial cancer lines were treated with increasing doses of Ln268. Cell proliferation rates were measured by Incucyte. Three independent biological replicates were performed for all the assays. All results are presented as the mean ± SD.

To further evaluate whether the suppressive effects of Ln268 are specific to Lin28-positive cancer cells, we compared cell growth rates of DuNE (Lin28b-positive line) and Du145 (Lin28b-negative line), both of which are Lin28a-negative. DuNE has the same genomic background with Du145 except for the transduction of the SRRM4 gene [4, 29]. We found that Ln268 had no impact on Du145 cell growth and cell morphology, in sharp contrast to its effects on DuNE cells (Fig. 5A). Consistently, we observed that Ln268 efficiently blocked DuNE spheroid growth in a time-dependent manner, but had no effect on Du145 spheroids (Fig. 5B). Furthermore, Ln268 had no impact on the cell growth of DuNE(KO) cells, in which the Lin28b gene was knocked out by CRISPR (Fig. 5C). However, Ln268 had strong suppressive effects to Lin28a-positive IGROV-1 cells (Fig. 5D). We also tested several ovarian and endometrial cancer cell lines with different Lin28a and Lin28b expression profiles (Fig. 5E). Ln268 suppressed the growth of Lin28a/Lin28b-positive TOV-112D, Kuramochi, IGROV-1, ANA3CA, and VOA1066 cells, but had no or minimal impacts to Lin28a/Lin28b-negative ES-2, HEC50, and HEC1B cells. Notably, the VOA1066 cells express both Lin28a and Lin28b proteins at relatively high levels and are extremely sensitive to Ln268 with an IC_50_ of 0.4 μM. These results demonstrate that the inhibitory effects of Ln268 to cancer cells are associated with Lin28a and/or Lin28b protein levels. They suggest that Ln268 has a broader therapeutic potential for various Lin28-positive tumors.

### Ln268 enhances stress inducers to inhibit cancer cell growth

**Fig. 6:**
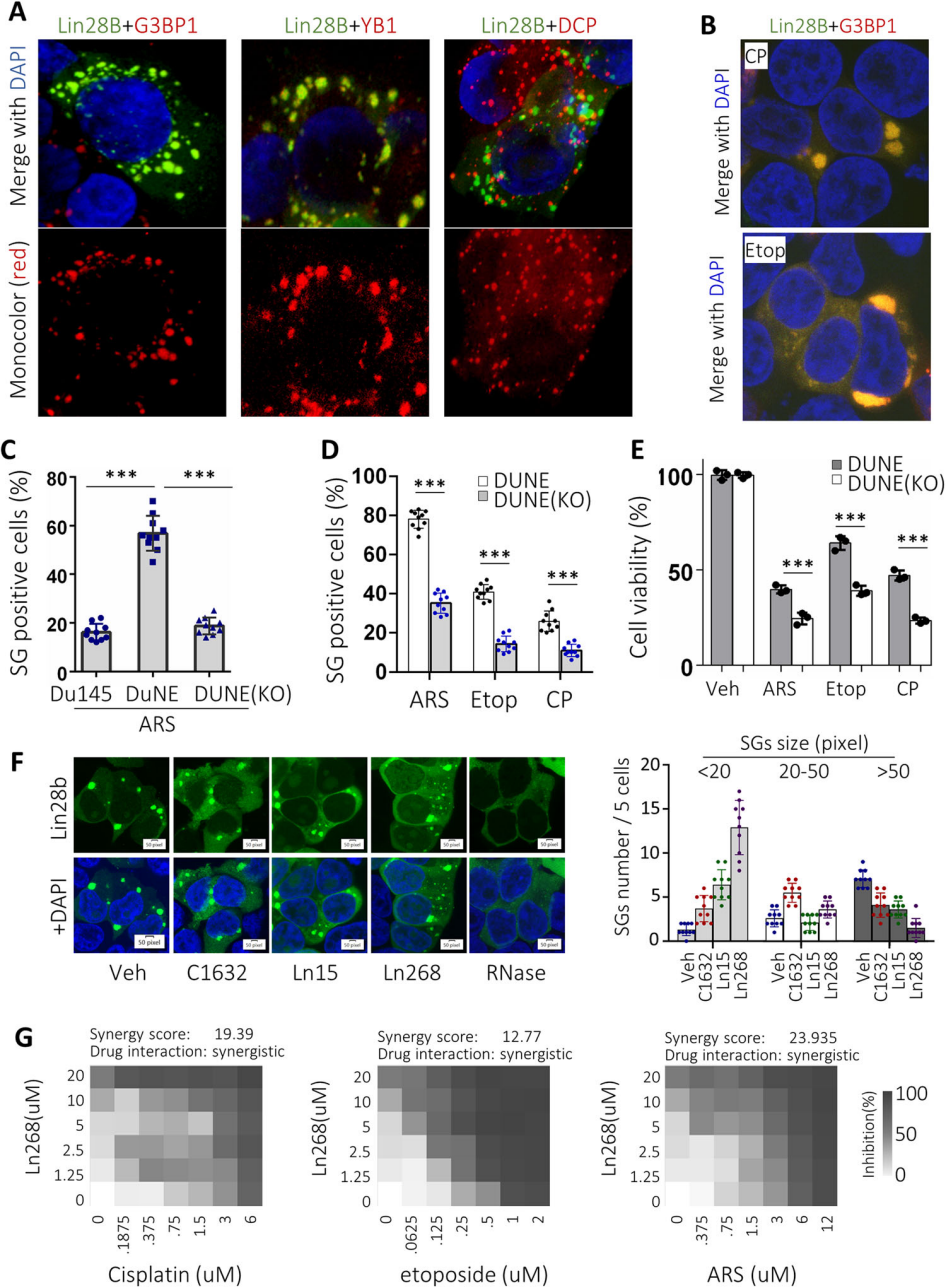
Ln268 enhances stress inducers to inhibit cancer cell growth. **A**, **B** DuNE cells were transfected with GFP-tagged Lin28b together with either RFP-tagged G3BP1, YB-1, or DCP1. Cells were then challenged with ARS (**A**), cisplatin (CP) or etoposide (etop) (**B**) to induce a stress condition. Confocal microscope detected the subcellular localization of Lin28b. **C**, **D** Du145, DuNE and DuNE(KO) cells were treated with ARS (**C**), cisplatin (CP) or etoposide (etop) (**D**). Percentage of cells forming SGs was counted in five random fields from two replicate experiments. **E** DuNE and DuNE(KO) cells were treated with ARS, cisplatin or etoposide. Cell viability was measured by Incucyte. **F** DuNE cells were transfected with GFP-tagged Lin28b and treated with ARS together with C1632, Ln15, Ln268, or RNase. Confocal microscope detected SGs formation mediated by Lin28b. Fluorescence images were captured, and SGs sizes and numbers were calculated as shown. **G** DuNE cells were co-treated with increasing concentrations of Ln268 plus cisplatin, etoposide and ARS. Drug interactions were calculated by SynergyFinder, with a synergy score higher than 10 representing synergistic relationship. Three independent biological replicates were performed for all the assays. All results are presented as the mean ± SD.

One molecular mechanism through which Lin28 regulates therapy-resistant tumor progression is by reprogramming mRNA translation. Under non-stress conditions, Lin28 is localized to polysomes where mRNAs are actively translated, and partially to P-bodies where RNAs are degraded [15]. However, under stress conditions, Lin28a is predominantly located in SG to sequester mRNAs away from polysomes to regulate selective mRNA translation, which function also relies on its RNA binding activity [15, 32]. Since SG formation is a defensive mechanism crucial for tumor cell survival [33, 34], Lin28 may also function to therapy-induced stress adaptation by promoting SG formation. Using DuNE cells to model therapy-induced neuroendocrine prostate tumors, we observed that Lin28b specifically localizes to SGs, as evidenced by its co-localization with SG markers G3BP1 and YB-1, but not with DCP1, a P-body biomarker, under stress conditions (Fig. 6A, B). Lin28b promotes SG formation, as its knockout dramatically reduces SG numbers induced by arsenite (ARS), etoposide, and cisplatin (Fig. 6C, D). Furthermore, Lin28b depletion renders DuNE cells more sensitive to ARS, etoposide and cisplatin treatments (Fig. 6E). These results demonstrate that loss-of-function of Lin28 could enhance the efficacy of chemotherapy drugs in suppressing tumor cells.

To assess whether Lin28 inhibitors can inhibit SG formation in cancer cells, we induced SGs in DuNE cells using ARS. These cells were co-treated with either vehicle, C1632, Ln15, or Ln268 (Fig. 6F). As a positive control, RNase was also included since it is known to prevent Lin28-mediated SG formation [15, 32]. Under vehicle treatment condition, Lin28b was primarily localized within SGs; however, in the presence of Lin28 inhibitors, its subcellular distribution became more diffuse, extending beyond SGs. SG formation involves a dynamic process in which initial smaller granules coalesce to form larger, mature SGs through phase separation (Fig. 6F). Notably, we observed that Lin28 inhibitors impeded the aggregation of smaller granules (<20 pixels) into larger SGs (>50 pixels), with Ln268 demonstrating the most suppressive effect. These findings indicated that Ln268 hinders the ability of Lin28b to promote SG formation.

We subsequently investigated whether Ln268 could potentiate anticancer effects of stress inducers to block cancer cell growth in vitro. First, we determined the IC_50_ values of each inhibitor, which were then used to formulate drug-drug interactions of Ln268 with cisplatin, etoposide, or ARS (Figure S5). DuNE cells were subjected to increasing doses of Ln268 in combination with cisplatin, etoposide, and ARS, as depicted (Fig. 6G). Ln268 exhibited synergistic effect with cisplatin, etoposide and ARS treatment, with synergy scores of 19.39, 12.77 and 23.93, respectively. These findings suggest that Ln268 can synergize with chemotherapy drugs to suppress tumor cell growth, whose effects were associated with its capacity to block Lin28 from promoting SG formation.

## Discussion

The integration of in silico drug screening with quantitative bioassays represents an efficient approach for discovering and designing novel inhibitors for protein targets previously deemed undruggable. This study exemplifies the targeting of the RNA-binding protein Lin28 through CADD, leading to the identification of a promising drug candidate, Ln268, for cancer therapy.

While RBPs such as Lin28 play crucial roles in tumorigenesis and therapy-resistant tumor progression, designing small molecules to block RBPs encounters many challenges. This is primarily due to the complex and non-catalytic nature of interactions between RBPs and RNA, as well as the high degree of intrinsically disordered regions within RBPs, which limits the discovery of specific inhibitors. The traditional approach involves high throughput assays such as FP and FRET, which screen thousands of compounds from a chemical library in a random manner to identify potential hits. However, this method is costly, time-consuming, and necessitates robotic instrumentation for automation. Despite these efforts, the success rate in typical large-scale industrial screens is less than 0.1% [18], many of which may be false positives. Moreover, when a candidate compound is identified, its MOA often remains unclear, impeding further drug development. Therefore, due to these intricate challenges posed by the complex nature of RBPs, particularly Lin28, the importance of rational small molecule design becomes increasingly evident.

In pursuit of this goal, we employed CADD, utilizing the crystal structure of the Lin28-RNA complex (PDB: 5UDZ [19]) and our previously identified scaffolds that target Lin28 [22]. Employing nucleobase-inspired design and SAR-guided strategies, we created 32 novel Lin28 inhibitors, of which 13 displayed activities in protein-based assays, indicating a 40% hit rate. Importantly, both strategies yielded active compounds—11/24 for SAR-guided and 2/8 for nucleobase guided. The discrepancies in hit rates between the two strategies could be explained by the fact that nucleobase-directed designed deviate more from the query scaffold.

The observed differences in the activity between closely related compounds revealed interesting SAR. For instance, the pairs of Ln250 derivatives—inactive Ln295 vs. active Ln298 and inactive Ln296 vs. Ln300—differ by one nitrogen atom added to Ln298 and Ln300, respectively, converting them into quinoline-like structures. The nitrogen atom in Ln298 and Ln300 introduces a different electronic distribution, making the ring system more electron-deficient and enhancing hydrogen bonding with the backbone of Tyr140 and the side chain of Asn141 (Figure S1). In another instance, the inactive compound Ln275, a bioisostere of Ln115, harbored an additional solvent-facing methyl group on the pyridinone ring, potentially affecting the compound’s water solubility or causing steric clashes with the charged surface of the Lin28 RNA-binding site, rendering the compound inactive. Similarly, inactive derivatives of Ln115, Ln277, and Ln282 also had modifications on the pyridinone ring, suggesting that this ring is sensitive to structural changes. However, the nucleobase-inspired design that modified the pyridinone ring into a purine-like base yielded two active compounds, Ln287 and Ln279. Together, SAR findings represent interesting directions for further optimization of lead compounds, such as modifying the quinoline-like structure to enhance electron deficiency or exploring nucleobase-inspired designs for improved RNA-binding affinity. Although these compounds showed promise in protein-based assays, not all passed the rigor of cell-based assays due to intricate interplays between protein affinity, cell permeability, metabolic instability, or off-target effects.

Ln267 and Ln268 are close analogs but exhibit distinct activity profiles in protein-based and cell-based assays. Ln267, featuring an additional hydroxyl group and an isopropyl group, displayed higher potency in FP assays, likely due to the formation of a hydrogen bond between Ln267 and Asn141, enhancing ligand-protein affinity. Its bulky isopropyl group, facing the solvent, may efficiently repel RNA. However, Ln267 possesses a larger topological polar surface area (TPSA) of 113.59 Å² and lower solubility (LogS of ~−4), predicted by SwissADME, compared to Ln15 and Ln268 (TPSA ~ 94 Å² and LogS ~ −3). These properties might hinder the cell permeability of Ln267, resulting in low effectiveness in cell-based assays. Conversely, Ln268, containing an electronegative fluorine atom that enhances cell permeability and membrane penetration, demonstrated strong activity in cell-based assays. However, the fluorine may decrease reactivity with the neighboring hydroxyl group, potentially weakening hydrogen bonding with Tyr140. This could explain why Ln268 performed less effectively than Ln15 in protein-based assays. These observations underscore the intricate interplay among structural modifications, reactivity, solubility, cell permeability, and activity profiles in both protein-based and cell-based assays. Future optimization of Ln268 should consider all perspectives to develop more potent Lin28 inhibitors.

Ln268 disrupts the capacity of Lin28 to promote SG formation, providing a rationale for a combination therapy involving the co-administration of Ln268 with chemotherapy drugs. SG formation serves not only as a common stress response in cancer cells, but also as an oncogenic mechanism that promotes tumor progression through CSC regulators. Unlike core SG proteins such as G3BP1, which indiscriminately bind various RNA species [35, 36], Lin28 selectively recognizes specific CSC gene transcripts to be sequestered in SGs under stress conditions [9, 37]. As Lin28-positive tumor cells exhibit a survival advantage in enduring chemotherapy-induced stress, this could lead to the enrichment of tumor cells with enhanced Lin28 signaling in therapy-resistant tumors. Our observations indicate that Ln268 synergizes with several stress inducers, including cisplatin, etoposide, and ARS, in suppressing tumor cell growth. This highlights a novel therapeutic strategy targeting Lin28-mediated SG formation in combination with chemotherapies.

In conclusion, we reported a new CADD campaign that led to the development of Ln268 as the most potent compound to block Lin28 from binding to its RNA substrates and inhibit Lin28 activity in multiple types of tumor cells. These findings pave the way for the development of new generation small molecule inhibitors for Lin28 target therapy.

## Materials and methods

### In silico modeling

Binding pose visualization and molecular modeling were performed using MOE software [38]. All the protein structures utilized for modeling, docking and molecular dynamics were prepared using Protein Preparation Wizard from Schrödinger [39]. The preparation included hydrogen bond assignment (using PROPKA with pH of 7.0), minimization with OPLS3e force field (with heavy atoms constrained to 0.30 Å RMSD) and addition of the missing side chains [40]. Ligand preparation was performed using the Omega [41] toolkit and included canonization of SMILES, ligand tautomerization and conformer generation. Molecular docking was performed with default parameters using GlideSP [39], FRED [42] and ICM [26] software; docking grids were calculated with the grid generation tools from respective software. Distances between docking poses were generated using MOE1 software. ADMET (absorption, distribution, metabolism, excretion, and toxicity) properties were calculated using SwissADME software [43]. A similarity search was performed with 3D ROCS and 2D GraphSim toolkits provided by OpenEye [44]. Derived analogs were then prepared the same way as the pre-docking preparation described above.

### Cells and chemicals

Du145 prostate cancer cell line was purchased from ATCC. It was used to generate DuNE cells and DuNE with Lin28b gene knockout by CRISPR as previously reported [4, 29]. IGROV-1, ES-2, Kuramochi, TOV-112D ovarian cancer cell lines and HEC50, HEC1B, AN3CA, VAO1066 endometrial cancer cell lines were generously provided Dr. Yanmin Wang from University of British Columbia. IGROV-1, ES-2, Kuramochi, TOV-112D, and HEC50 were cultured in RPMI-1640 with 10% Fetal Bovine Serum (FBS). DuNE, Du145, HEC1B, AN3CA, VAO1066 were cultured in DMEM with 10% FBS. All the cell lines were incubated in 5% CO_2_ at 37 °C and they tested negative for mycoplasma contamination and were authenticated by short tandem repeat assays. All chemicals were custom synthesized by Life Chemicals Ltd. with a purity of >90%. LC/MS/MS was used to confirm the molecular masses and purity of the as-received compounds.

### Standard biochemistry and molecular techniques

Several standard biochemistry and molecular methods including FP, electrophoresis mobility shift assays (EMSA), cell proliferation assays, real-time qPCR, colony formation, and immunoblotting assays had been described in detail in our previous publications [22, 45, 46]. This information can also be found in the Supplementary Materials and Methods section attached to this manuscript.

### Spheroid formation

For a spheroid generation, 1000 cells/well were seeded in 96-well ULA round-bottomed plates (Corning, Amsterdam) using a multichannel pipette. 3D multicellular spheroids were spontaneously generated after 24 h culture. Plates were incubated for 6 days at 37 °C, 5% CO_2_, and 95% humidity. DuNE and Du145 cells were cultured in DMEM with 10% FBS. Fully automated image analysis of tumor spheroids was carried out with the Incucyte® S3 Live-Cell Analysis System. The size and shape of multicellular spheroids were analyzed by using the Incucyte® Spheroid Analysis Software Module.

### Nuclear magnetic resonance (NMR) spectroscopy

NMR spectra were recorded at 5 °C on a Bruker Avance III 850 MHz spectrometer, equipped with a TCI cryoprobe. ^1^H-^15^N HSQC experiments [47] were recorded on ^13^C/^15^N-labeled Lin28b ZKD (230 μM), in 20 mM d-Tris-HCl (pH 7.5), 100 mM NaCl, 1 mM d-DTT, 0.02% NaN_3_, 0.25 mM ZnCl_2_ + 1 mM IDA, and 10% D_2_O, to which unlabeled Ln15 and Ln268 was added, respectively. The time domain matrix was 4k by 512. Four scans were acquired per t1 increment. Spectra were processed and plotted using Topspin to a final size of 8k by 1k. For the overlays, the levels were set to the same base level in all cases, with a multiplication factor of 1.1. Sixteen levels are displayed.

### Statistics

For statistical analysis, we utilized the GraphPad Prism 9.01 software (GraphPad Software, San Diego, CA, USA). Using a student t-test, the differences between the two groups were compared. One-way ANOVA was used to compare differences across several groups, and a t-test was used after that. The thresholds for significance were chosen at *p* < 0.05 as *, *p* < 0.01 as **, and *p* < 0.001 as ***, respectively.

## Supplementary information

uncropped images

suppl file

## Data Availability

The datasets generated during and/or analyzed during the current study are available from the corresponding author on reasonable request.
