# Supplementary material for: Developing novel Lin28 inhibitors by computer aided drug design
Source: Cell Death Discov. 2025 Jan 12;11:5. doi: 10.1038/s41420-024-02281-z (PMC11725581; doi:10.1038/s41420-024-02281-z)

Original Images
Figure 2

C

Let-7 probe titration | Lin28b ZKD titration | Let-7 competitor titration

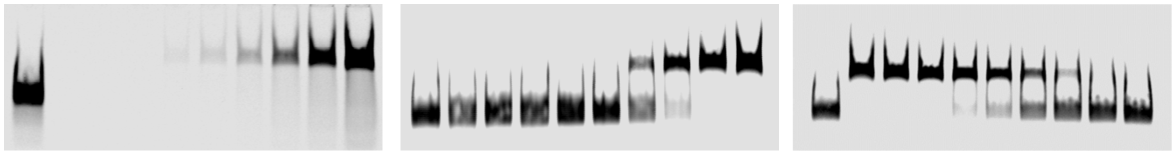

D

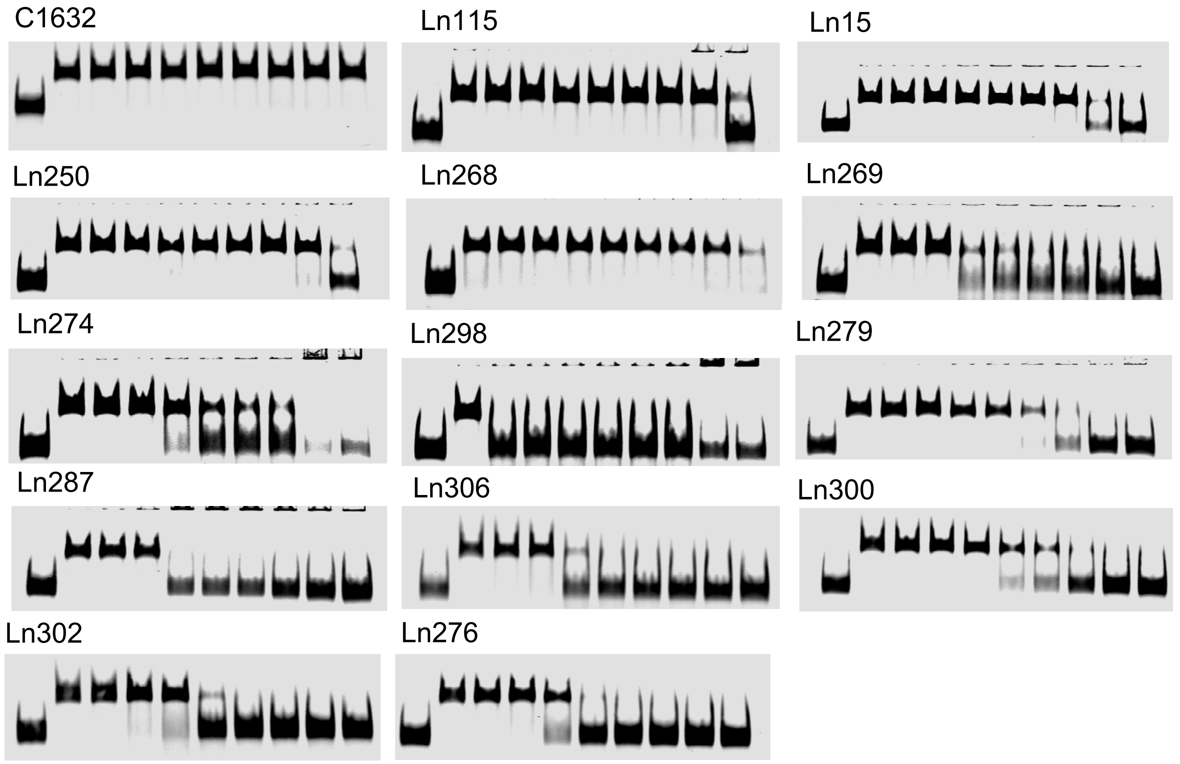

Original Images
Figure 3

B

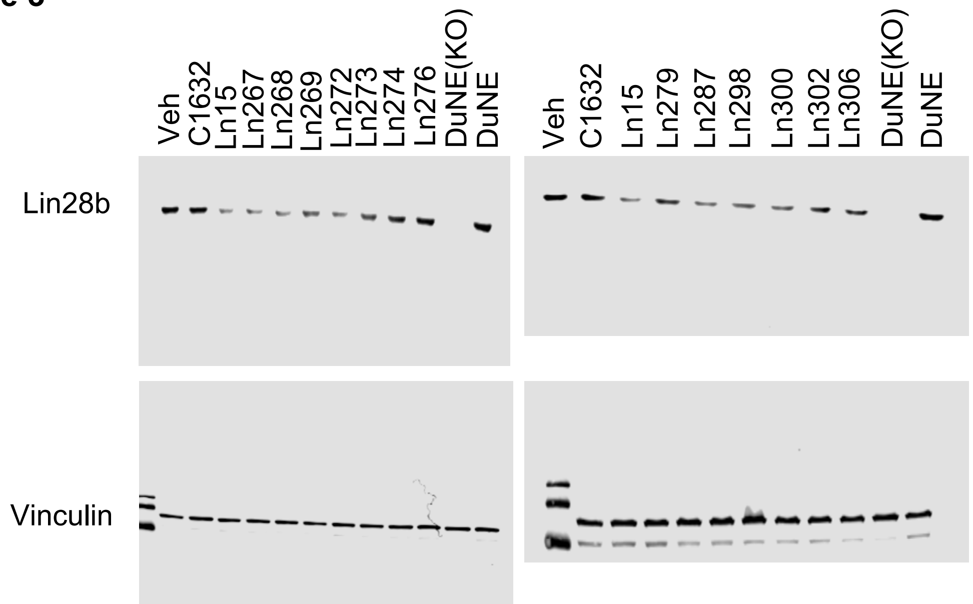

Original Images Figure 4

Original Images Figure 5

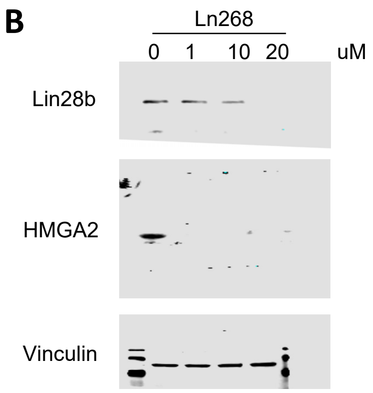

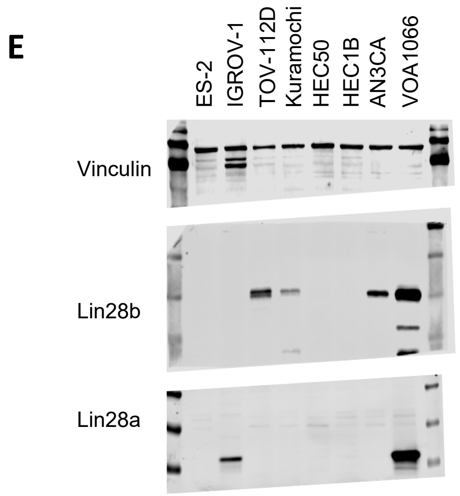

Original Images Figure S6

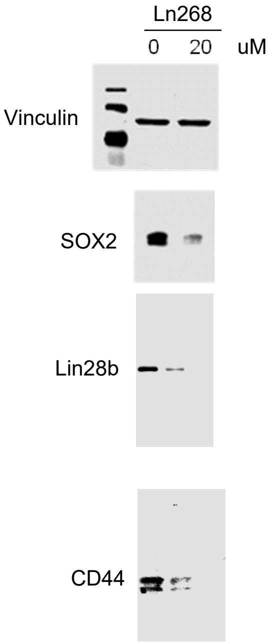

Supplement: Supplementary file 1 — uncropped images [file 41420_2024_2281_MOESM1_ESM.pdf]
