# Supplementary material for: Developing novel Lin28 inhibitors by computer aided drug design
Source: Cell Death Discov. 2025 Jan 12;11:5. doi: 10.1038/s41420-024-02281-z (PMC11725581; doi:10.1038/s41420-024-02281-z)
Supplement: Supplementary file 2 — suppl file [file 41420_2024_2281_MOESM2_ESM.docx]

**Developing novel Lin28 inhibitors by computer aided drug design**

Victor M. Matias-Barrios ^1, *^, Mariia Radaeva ^1, *^, Graciella Rosellinny ^1^, Qiongqiong Jia ^1^, Ning Xie ^1^, Monica Villanueva^2^, Hanadi Ibrahim^1^, Jason Smith ^1^, Martin Gleave^1^, Nada Lallous ^1^, Suzana K. Straus ^2^, Artem Cherkasov ^1^, Xuesen Dong ^1, 3^

1. **Supplementary Materials and Methods**
2. **Supplementary Figures**
3. **Supplementary Methods**

**Fluorescence Polarization (FP) Assay**

The fluorescein amidites (FAM)-tagged pre-Let-7d probe with the sequence of UUAGGGCACGAGAUUUUGCCCACAAGGAGUU was purchased from Integrated DNA Technologies (IDT). The probe at the concentrations ranging from 1 nM to 50 uM was incubated with Lin28B (ZKD) or Lin28 (CSD+ZKD) to a final volume of 30 uL at room temperature for 20 min. To read the fluorescence, a TECAN 500 machine was used. Fluorescence polarization was calculated using the following equation:

𝑃=(𝐹𝑝𝑎𝑟𝑟𝑎𝑙𝑙𝑒𝑙−𝐹𝑝𝑒𝑟𝑝𝑒𝑛𝑑𝑖𝑐𝑢𝑙𝑎𝑟)/(𝐹𝑝𝑎𝑟𝑟𝑎𝑙𝑙𝑒𝑙+𝐹𝑝𝑒𝑟𝑝𝑒𝑛𝑑𝑖𝑐𝑢𝑙𝑎𝑟𝑃)

where *P* is the fluorescence polarization reading, *F parallel* is the fluorescence intensity acquired from the parallel excitation plane, and *F perpendicular* is the fluorescence intensity acquired from the perpendicular direction of the excitation plane. The total fluorescence intensity is estimated using the following equation:

𝐹=𝐹𝑝𝑎𝑟𝑎𝑙𝑙𝑒𝑙+2𝐹𝑝𝑒𝑟𝑝𝑒𝑛𝑑𝑖𝑐𝑢𝑙𝑎𝑟𝐹.

All the raw FP signal data from the TECAN 500 FP results were analyzed using Prism 8.0 (GraphPad, San Diego, CA, USA); normalized curves were plotted and IC50 values were estimated. All FP data were repeated three to five times.

**Electrophoresis mobility shift assay (EMSA)**

EMSA followed the standard protocols we published [1]. An IRDye800CWN-labeled pre-Let-7d RNA probe (5’-IRD800CWN-UUAGGGCAGAGAUUUUGCCCACAAGGAGUU-3’) was purchased from IDT (Coralville, IA, USA). Lin28a or Lin28b proteins were incubated with 300 nM of probe in a standard 20 uL EMSA reaction in a reaction buffer containing 20 mM HEPES-NaOH, pH 7.4, 2 mM DTT, 3 mM MgCl_2_, 0.05% NP-40, protease inhibitors, and 0.1 ug poly[dI:dC]. After incubation for 30 min at 37 °C, reactions were loaded onto 4% non-denatured polyacrylamide gel in 0.5× TBE buffer. The RNA oligo bands were visualized by using the Li-Cor Odyssey 9120 Infrared Imaging System.

**Real-time PCR and immunoblotting assays**

Total RNA was extracted by TRIZOL reagent (Invitrogen), treated with deoxyribonuclease and reversely transcribed by random hexamers and superscript II (Invitrogen). Real-time qPCR was performed on the ABI PRISM 7900 HT system (Applied Biosystems) using the FastStart Universal SYBR Green Master mix (Roche) following standard protocol as we reported [2]. All real-time qPCR assays were carried out in triplicates from three independent cDNA syntheses. For immunoblotting assays, whole cell lysis was extracted by a buffer containing 50mM of Tris pH8.0, 150mM of NaCl, 1% NP40, 0.5% sodium deoxycholate, 0.1% SDS and proteinase and phosphatase inhibitors (Roche). Protein lysis separated by SDS-PAGE, transferred to polyvinylidene difluoride membrane, and blotted by specific antibodies [3-5].

**Table S1. Real time-qPCR Primer Information**

| **Primer Name** | **Forward Primer Sequence (5’-3’)** | **Reverse Primer Sequence (5’- 3’)** |
| --- | --- | --- |
| Lin28b | TGTAGTCTACCTCCTCAGCCAA | ATTCTGCTTCCTGTCTTCCCTG |
| HMGA2 | AGTCCCTCTAAAGCAGCTCAAAAG | GCCATTTCCTAGGTCTGCCTC |
| IGF2BP1 | GGCCATCGAGAATTGTTGCAG | CCAGGGATCAGGTGAGACTG |
| OCT4 | GTGTTCAGCCAAAAGACCATCT | GGCCTGCATGAGGGTTTCT |
| Nanog | TTTGTGGGCCTGAAGAAAACT | AGGGCTGTCCTGAATAAGCAG |
| ID4 | GGCCACTCAAGCAGCATTTG | TCTGGTTGCCTGGTTAGGAC |
| FoxC1 | TGTTCGAGTCACAGAGGATCG | ACAGTCGTAGACGAAAGCTCC |
| ALDH1A2 | TTGCAGGGCGTCATCAAAAC | ACACTCCAATGGGTTCATGTC |
| FoxO3 | TCACGCACCAATTCTAACGC | CACGGCTTGCTTACTGAAGG |
| CHGA | TAAAGGGGATACCGAGGTGATG | TCGGAGTGTCTCAAAACATTCC |
| CHGB | CGAGGGGAAGATAGCAGTGAA | CAGCATGTGTTTCCGATCTGG |
| SYP | TTAGTTGGGGACTACTCCTCG | TTAGTTGGGGACTACTCCTCG |
| SCGN | GGCCATTTCTGAGGCTAAACT | GGGCTCCTGTTTTACTAACATCA |
| Let-7d | CCAGCTGGGAGAGGTAGTAGGTTGC | CTGGTGTCGTGGAGTCGGCAATT |
| **Primer Name** | **Stem Loop Primer Sequence (5’-3’)** | |
| Let-7d | CTCAACTGGTGTCGTGGAGTCGGCAATTCAGTTGAGAACTATGC | |
| U6 | CTCAACTGGTGTCGTGGAGTCGGCAATTCAGTTGAGAAAAATATG | |

**Table S2. Antibody information**

| **Antibody** | **Vendor** | **Catalogue number** | **Application** | **Dilution** |
| --- | --- | --- | --- | --- |
| Lin28a | Abcam | Ab124765 | WB, IF | 1:1000 |
| Lin28b | Abcam | Ab71415 | WB | 1:1000 |
| HMGA2 | Thermo Fisher | PA5-21320 | WB | 1:1000 |
| SOX2 | Cell Signaling | 3579S | WB | 1:1000 |
| CD44 | eBioscience | 14-0441-82 | WB, FC, IF | 1:500 |
| Vinculin | Sigma Aldrich | V9131-2ML | WB | 1:1000 |

FC = Flow Cytometry IF = Immunofluorescence WB = Western blot

**Cell proliferation and FACS**

Cell proliferation rates were measured by using Incucyte® Live-Cell Analysis System (Sartorius) following the manufacturer's protocol. Cell cycling was assessed by using BrdU incorporation into S-phase DNA by using the APC BrdU flow kit (BD Pharmingen; Franklin Lakes, New Jersey) according to the manufacturer’s protocol. One mM of BrdU was added to cells and incubated for 6 hours. Cells were incubated with anti-BrdU antibody and stained with 7-AAD before processing the cells for flow cytometry [3].

**Colony formation**

In the colony formation assays, cancer cells were seeded in 0.7% soft agar in a 6-well plate, with a 1% soft agar bottom base coating. Cells were allowed to grow for 14 days to form colonies. Colonies were stained with crystal violet and imaged by stitching 5X field images together to capture the entire well (Zeiss light microscope [Carl Zeiss]). Colony numbers were counted according to their diameters (100-300um or >300um). Three independent biological replicates were performed for all the assays.

**Confocal microscopy**

Cells were fixed with 4% paraformaldehyde, treated in 0.25% Triton X-100 for 15 mins, and mounted with DAPI staining mount (Vector Labs; Burlingame, USA). Cell images were captured by confocal microscopy at 63X magnification (Zeiss; Germany) [4, 5].

**Statistics**

Statistical analysis was performed using the GraphPad Prism 5.01 software (GraphPad Software, CA, USA). Differences between the two groups were compared by student t-test. One-way ANOVA followed t-test was used to compare differences among multiple groups. The levels of significance were set at p<0.05 as *, p<0.01 as **, and p<0.001 as ***.

**Reference**

1. Radaeva, M., et al., *Discovery of Novel Lin28 Inhibitors to Suppress Cancer Cell Stemness.* Cancers (Basel), 2022. **14**(22).

2. Yu, Y., et al., *Expression and function of the progesterone receptor in human prostate stroma provide novel insights to cell proliferation control.* J Clin Endocrinol Metab, 2013. **98**(7): p. 2887-96.

3. Lee, A.R., et al., *A novel mechanism of SRRM4 in promoting neuroendocrine prostate cancer development via a pluripotency gene network.* EBioMedicine, 2018.

4. Li, H., et al., *UGT2B17 Expedites Progression of Castration-Resistant Prostate Cancers by Promoting Ligand-Independent AR Signaling.* Cancer Res, 2016. **76**(22): p. 6701-6711.

5. Li, H., et al., *Catalytic inhibitors of DNA topoisomerase II suppress the androgen receptor signaling and prostate cancer progression.* Oncotarget, 2015. **6**(24): p. 20474-84.

1. **Supplementary Figures**

**Figure S1.** Lin28b ZKD protein was incubated with the indicated compounds at the concentration of 0, 0.1, 0.2, 0.5, 1, 2, 5, 10, 20, 50, 100, and 200uM. FP signals were measured and plotted relative to that from vehicle.


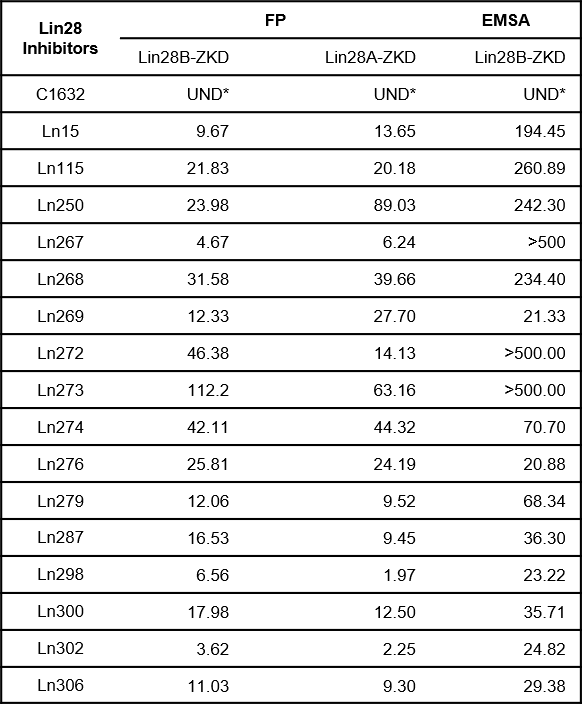


**Table S1 FP and EMSA IC50 estimation**

***UND = undetermined**

**
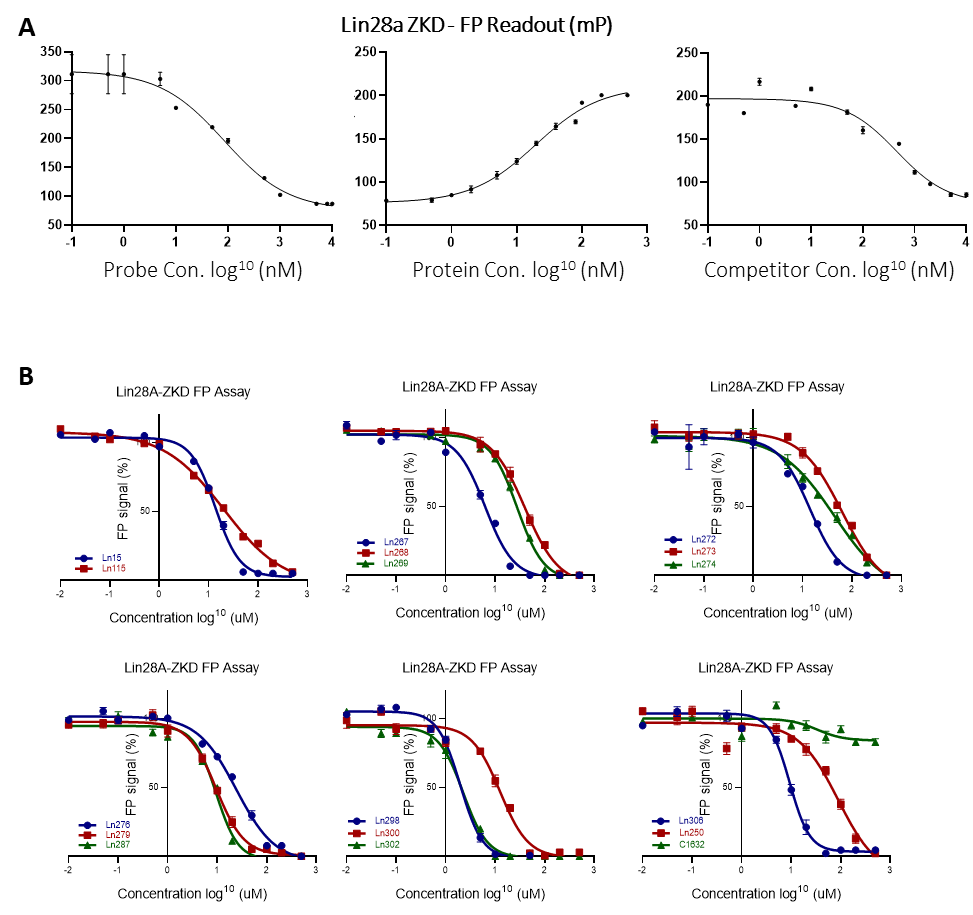
**

**Figure S2**. Lin28a ZKD protein was incubated with the indicated compounds at the concentration of 0, 0.1, 0.2, 0.5, 1, 2, 5, 10, 20, 50, 100, and 200uM. FP signals were measured and plotted relative to that from vehicle.


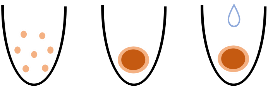

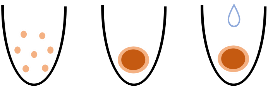

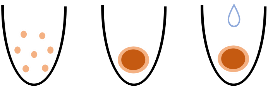


Cell

seeding

**(Day -2)**

Spheroid

formation

**( Day -1)**

Add

treatment

**(Day 0)**

96 ultra-low attachment plate

growth monitoring with Incucyte

Days

0

2

4

6

DuNE


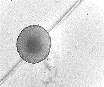

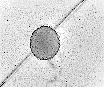

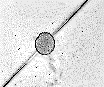

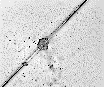


Spheroid volume

(uM^3^)x10^6^

1.5

1.0

0.5

0

0

2

4

6

Days


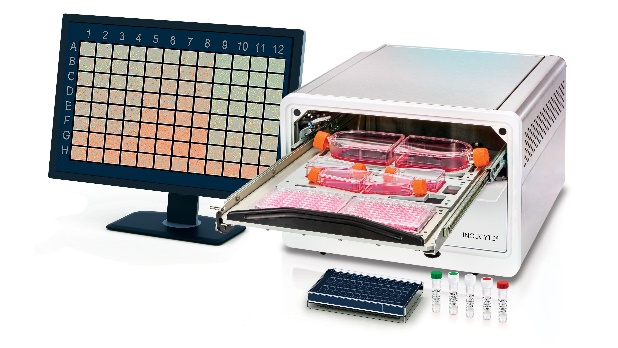


**Figure S3.** To establish prostate cancer cell spheroid model**, c**ancer cells were seeded in low attachment round bottom wells for two days to allow spheroids to form. Spheroid sizes will be captured over time by Incucyte, and plotted as a growth curve.


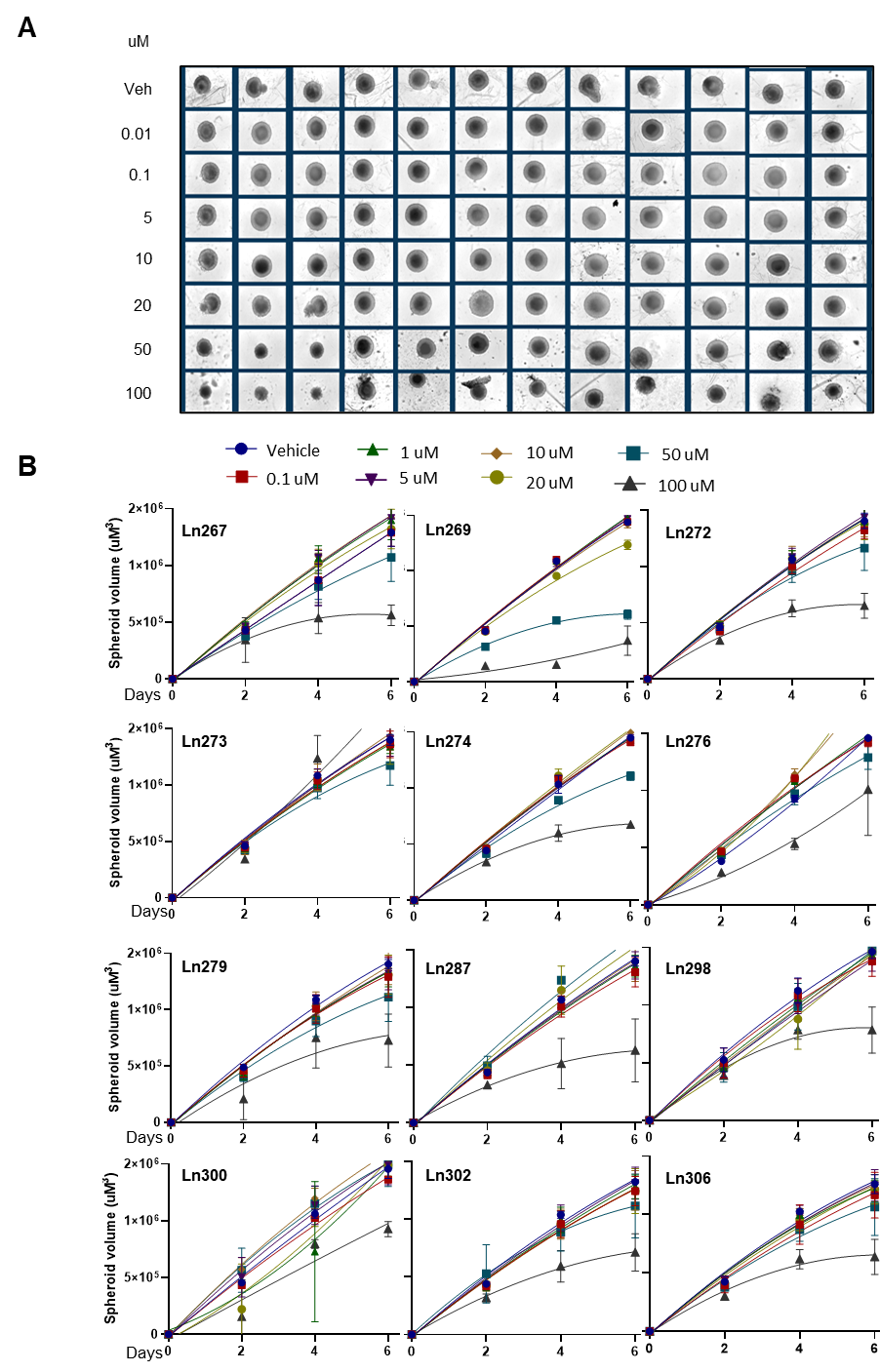


**Figure S4.** Time- and concentration-dependent effect of Lin28 inhibitor derivatives on DuNE spheroids growth. A) Representative bright-field images at day 6 of spheroids treated with Lin28 inhibitors are shown. B) Time-related volume of DuNE tumor spheroids after treatment with Lin28 inhibitors at the codependence time and concentration.

| **cell** | **Drug** | **IC50(nM)** |
| --- | --- | --- |
| DuNE | ARS | 2734 |
|  | Cisplatin | 1523 |
|  | Etoposide | 543 |
|  | Ln268 | 6286 |

**Figure S5** DuNE cells were treated with increasing doses of ARS, cisplatin, etoposide and Ln268 to determine the IC50 values, which were used to design experiments to study drug-drug interactions by SyngeryFinder.


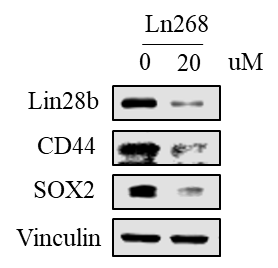


**Figure S6.** DUNE cells were treated with 20uM of indicated compounds for 72 hours. Protein lyses were collected and immunoblotted with Lin28b, CD44, and SOX2 antibodies. Vinculin was used as the loading control.
